# Pangenome analysis reveals transposon-driven genome evolution in cotton

**DOI:** 10.1186/s12915-024-01893-2

**Published:** 2024-04-23

**Authors:** Xin He, Zhengyang Qi, Zhenping Liu, Xing Chang, Xianlong Zhang, Jianying Li, Maojun Wang

**Affiliations:** National Key Laboratory of Crop Genetic Improvement, Hubei Hongshan Laboratory, Huazhong Agricultural University, Wuhan, China

**Keywords:** Cotton, Comparative pangenome, LTR retrotransposon, Polyploidy

## Abstract

**Background:**

Transposable elements (TEs) have a profound influence on the trajectory of plant evolution, driving genome expansion and catalyzing phenotypic diversification. The pangenome, a comprehensive genetic pool encompassing all variations within a species, serves as an invaluable tool, unaffected by the confounding factors of intraspecific diversity. This allows for a more nuanced exploration of plant TE evolution.

**Results:**

Here, we constructed a pangenome for diploid A-genome cotton using 344 accessions from representative geographical regions, including 223 from China as the main component. We found 511 Mb of non-reference sequences (NRSs) and revealed the presence of 5479 previously undiscovered protein-coding genes. Our comprehensive approach enabled us to decipher the genetic underpinnings of the distinct geographic distributions of cotton. Notably, we identified 3301 presence-absence variations (PAVs) that are closely tied to gene expression patterns within the pangenome, among which 2342 novel expression quantitative trait loci (eQTLs) were found residing in NRSs. Our investigation also unveiled contrasting patterns of transposon proliferation between diploid and tetraploid cotton, with long terminal repeat (LTR) retrotransposons exhibiting a synchronized surge in polyploids. Furthermore, the invasion of LTR retrotransposons from the A subgenome to the D subgenome triggered a substantial expansion of the latter following polyploidization. In addition, we found that TE insertions were responsible for the loss of 36.2% of species-specific genes, as well as the generation of entirely new species-specific genes.

**Conclusions:**

Our pangenome analyses provide new insights into cotton genomics and subgenome dynamics after polyploidization and demonstrate the power of pangenome approaches for elucidating transposon impacts and genome evolution.

**Supplementary Information:**

The online version contains supplementary material available at 10.1186/s12915-024-01893-2.

## Background

Cotton produces the world’s most important natural textile fibers and serves as a model system for studying plant polyploidization. Approximately 1–2 million years ago (MYA), two diploid progenitors (genome types AA and DD) underwent natural hybridization and chromosome doubling, giving rise to the formation of allotetraploid cotton (AADD) [1, 2]. Two cultivated allotetraploid species, *Gossypium hirsutum* (AD)_1_ and *Gossypium barbadense* (AD)_2_, have been domesticated by humans and have dominated modern cotton breeding. Compared with diploid ancestral progenitors, allotetraploid cotton gained numerous genomic variations, including chromosome rearrangement, gene silencing, and epigenetic changes [3–6].

As cotton genomics has advanced, comparative genome analysis has been used to study genomic structure differences between tetraploid and diploid progenitors. By combining PacBio or Oxford Nanopore Technologies (ONT) long-read sequencing with high-throughput chromosome conformation capture data, the reference genomes of *G. arboreum* (A_2_), *G. raimondii* (D_5_), and *G. hirsutum* (AD)_1_ were released [7, 8]. LTR retrotransposons are major components of most larger plant genomes, arising through a “copy-and-paste” mechanism, and are known to be responsible for the remarkable variation in genome size diversity and species differentiation within the *Gossypium* genus [9]. Previous studies have identified extensive genomic variations and changes in transposable element (TE) content between diploid and tetraploid cotton. When LTRs occur within gene exons, they are often disruptive, but sometimes new genes are produced by TE insertion [10, 11]. Moreover, amplification divergence of LTR retrotransposons between tetraploid and diploid cotton led to a decrease in the A_t_ (with the lowercase “t” denoting tetraploid) (A_t_ ~ 1400 Mb vs. A_1_ or A_2_ ~ 1621 Mb) but an increase in the D_t_ (D_t_ (~ 796 Mb vs. D_5_ ~ 750 Mb) subgenome [12].

Due to genomic diversification, previous studies based on a single reference genome missed abundant genomic variation and failed to comprehensively detect sequence diversity. To solve this shortcoming, researchers proposed the concept of the pangenome, including the core and dispensable genomes [13]. Ideally, the pangenome captures all genetic variations within a species. Several pangenomes have been constructed in plants, such as rice, tomato, wheat, soybeans, chickpea, and tetraploid cotton, which have improved our understanding of genetic variation, species diversity, and key genes associated with agronomic traits [14–17]. A pangenome comparison between the allopolyploid *B. hybridum* and its diploid progenitors *B. distachyon* and *B. stacei* revealed a gradual accumulation of small variations and a constant loss of genes after polyploidization [18]. In allopolyploid soybean, a study found the accumulation of small deletions in gene clusters through illegitimate recombination [19]. Comparison of allopolyploid *B. napus* with its diploid progenitor *B. oleracea* revealed gene loss resulting from TE activity in diploids. However, in tetraploids, the gene loss was associated with chromosome location [20]. In cotton, a comparative pangenome analysis between tetraploid and diploid has yet to be reported.

Here, we constructed a pangenome of diploid A-genome cotton, which provided valuable insights into its evolutionary features, specifically focusing on the role of transposons in gene evolution and the effects of polyploidization. We identified 511 Mb of novel NRSs, discovered 5479 previously unknown protein-coding genes, and verified the influence of LTR retrotransposons in shaping the genome structure. Through comparisons among different cotton species, we observed variations in genome composition following polyploidization, revealing distinct evolutionary trajectories between diploid and tetraploid species. We found that TE activity was responsible for gene loss and the generation of species-specific genes.

## Results

### Pangenome construction of a diploid A-genome cotton

We collected a total of 344 accessions to represent the diploid cotton A-clade, comprising 29 accessions of *Gossypium herbaceum* (A_1_) and 315 accessions of *Gossypium arboreum* (A_2_), with an average genome sequencing coverage of 24.1 × , which contain all the published diploid A-genome cotton resequencing data. The accessions were obtained from diverse regions, including 222 accessions from China (CHN), 52 from South Asia, 26 from the United States (US), and 15 with undetermined geographic origins (Additional File 2: Table S1). We used the “map-to-pan” approach to construct the diploid A-clade pangenome. For each accession, we performed de novo assembly and retained contigs longer than 500 bp; then, the contigs were aligned to the Shixiya 1 reference genome to identify the NRSs. After excluding redundant and potential contaminating sequences, we finally obtained 511 Mb of NRSs and integrated them with the “Shixiya1” reference genome to yield the diploid A-clade pangenome of 2166 Mb [8].

We evaluated the quality of the pangenome by three approaches. First, we observed an improvement in the mapping rate of short reads from 344 accessions, which increased from 98.5 to 99.7% from the reference to the pangenome. Second, we evaluated the completeness of BUSCO hits using the eudicots_odb10 database and found 97.7% and 98.9% completeness in the reference and pangenomes, respectively (Additional File 1: Fig. S1a). Third, we aligned the 511 Mb NRSs to the nucleotide collection (nt) database and found that 95.8% of NRSs had alignments in the *Gossypium* genus (Additional File 1: Fig. S1b). These results indicate high confidence in the pangenome.

We annotated a total of 5479 non-reference genes on the NRSs, of which 75.2% (4123) were assigned gene functions through the Gene Ontology (GO) (47.5%), Kyoto Encyclopedia of Genes and Genomes (KEGG) (39.2%), and Pfam (49.8%) databases. Finally, the diploid cotton pangenome was 2166 Mb with 47,257 genes (1655 Mb with 41,778 genes for the reference genome) (Additional File 2: Table S2).

**Fig. 1:**
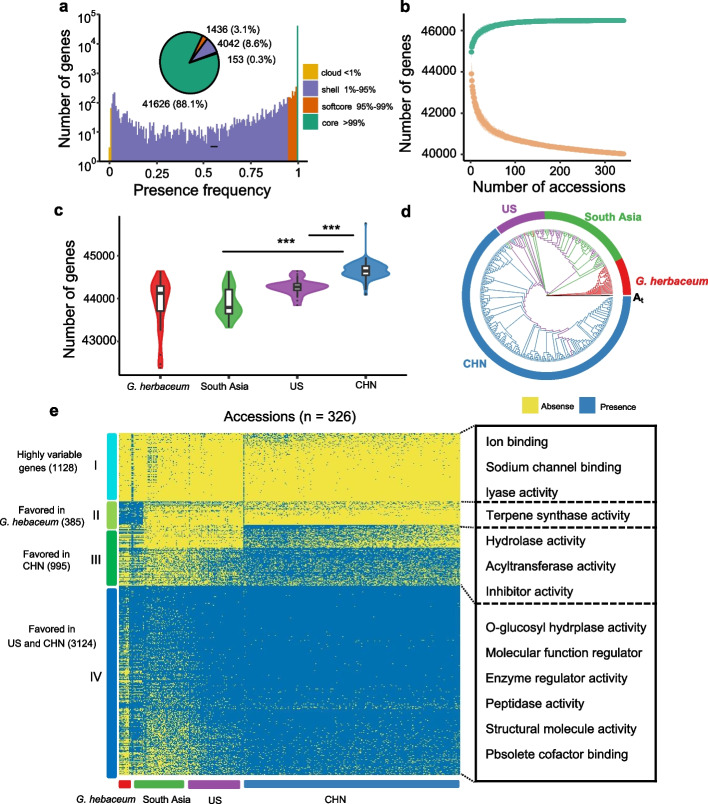
Pangenome of *Gossypium arboreum*. **a** The gene number and presence composition of the *G. arboreum* pangenes. Pie charts correspond to the proportions of the core, soft core, shell, and cloud genes according to gene presence in the population. **b** The modeling analysis of the number of pangenes and core genes in 341 cotton accessions. The top and bottom edges represent 99% confidence intervals. **c** The boxplot displays the number of genes in each group. *P-*values (“***” indicate *P* <  0.01) were calculated by a two-sided Mann–Whitney *U* test. **d** Maximum-likelihood tree of the 326 known geographic accessions was constructed using the 5632 dispensable genes. **e** Heatmap showing the PAVs of 5632 dispensable genes in 326 accessions. K-means (*K* = 4) clustering was used to cluster genes. Each cluster is shown with one or two geographical distributions, and enriched GO terms in each cluster are displayed in the right panel

Presence and absence variations (PAVs) in genes among the different accessions can reveal genetic changes and the breeding history. After excluding three outgroup accessions, 47,257 pangenes from 341 accessions were used for gene PAV analysis. We identified 41,626 (88.1%) core genes, 1436 (3.0%) soft core genes, 4042 (8.6%) shell genes, and 153 (0.3%) cloud genes, which were present in more than 99% (> 337), 95–99% (324–337), 1–95% (4–323), and less than 1% (< 4) of accessions, respectively (Fig. 1a; Additional File 1: Fig. S2). To verify the accuracy of gene PAVs, a PCR experiment of five dispensable genes in 23 randomly selected accessions matched the results from the bioinformatics analysis (Additional File 1: Fig. S3; Additional File 2: Table S3), further confirming the reliability of the gene PAV data and providing confidence in the pangenome.

We explored the associations between the gene PAVs and genic features, encompassing Pfam domain annotation, expression level, exon number, gene length, selective constraint, and TE coverage. The result demonstrated a significant association between gene PAVs and the investigated genic features (Additional File 1: Fig. S4). Specifically, dispensable genes were more likely to be evolving under relaxed selective constraint compared to core genes, indicating that the evolution rate of dispensable genes was faster than that of core genes, consistent with previous findings in rice, tomato, and wheat [14, 15, 21]. Our analysis also showed a closed pangenome, and we gradually increased the number of selected samples to estimate the size of the pangenes and core genes (Fig. 1b), indicating that the sampling strategy in this study covered the genetic diversity in diploid A-clade cotton.

The gene PAV-based phylogenetic analysis showed that the PAVs were broadly distributed within different subpopulations. Interestingly, we observed that the Chinese (CHN) group encoded more genes than the South Asian and US groups (Fig. 1c), indicating that *G. arboreum* migrated to high latitudes and formed distinctive genes after long-term selection. Moreover, we identified a small clade of US accessions that were clustered with accessions from South Asia and CHN in the phylogenetic tree (Fig. 1d), suggesting that some US accessions were genetically mixed with accessions from South Asia and China. We further tabulated the PAV genes in four subpopulations. The k-means algorithm classified the 5632 dispensable genes into four clusters. Each cluster enriched one or two geographic origins, and accessions with different geographic origins had clear divergence. In cluster I, we found that 1128 genes had a lower presence frequency in 326 accessions, which were enriched in the gene functions of ion transport and sodium channel binding and lyase activity. The 385 genes in cluster II were mainly present in *G. herbaceum* and were involved in terpene synthase activity. The 995 genes in cluster III tended to be of CHN origin, which was involved in hydrolase activity and acyltransferase activity. Cluster IV contained 3124 genes that tended to be of both CHN and US origin, which were involved in basic biological processes (Fig. 1e). To investigate signals of gene frequency changes between the CHN and non-CHN populations, we conducted comparisons based on the dispensable genes. Genes exhibiting a substantial frequency change (fold change > 2 or < 0.5; FDR < 0.001) were categorized as either favorable or unfavorable in the CHN group. Specifically, we identified 645 genes with high frequency and 103 genes with low frequency in the CHN group. GO analysis unveiled that the functions of CHN favorable genes were enriched in cellulose synthase activity and terpene synthase activity. Low-frequency genes were enriched in channel activity, indicating potential implications in maintaining cellular homeostasis, signal transduction, and various physiological processes (Additional File 1: Fig. S5).

### The pangenome improves eQTL detection

**Fig. 2:**
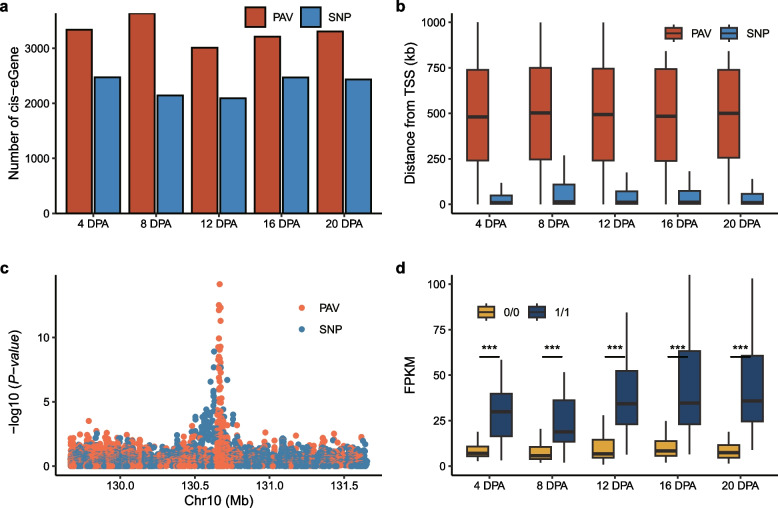
eQTL analysis based on the pangenome and enhanced discovery of causal variation. **a** Bar plot showing significant eQTLs identified by PAVs and SNPs at different fiber developmental stages. **b** Distance of the significant variation from the TSS of the associated genes. **c** An example of a PAV lead-eQTL for the Garb_10G032080 gene. The *y*-axis represents the significance of the association. **d** Boxplot of the gene expression levels of different PAV alleles at different fiber developmental stages

It has been suggested that PAVs are more likely than SNPs to alter gene expression [22]. In this study, we identified 725,998 PAVs in NRSs. Then, we integrated the RNA-seq data from 216 accessions at 5 fiber development timepoints and SNP-PAV variations to perform expression quantitative trait locus (eQTL) analysis. We identified an average of 3301 best *cis*-PAV-gene pairs with significant expression associations with a false discovery rate (FDR) less than 0.05 at each timepoint, compared with an average of 1942 *cis*-SNP-gene pairs (Fig. 2a), which showed a strong link between PAVs and gene expression regulation during fiber development. For these, we compared the results from previous research and found that an average of 2342 *cis*-PAV genes were novel in each period [23]. In addition, we observed that PAVs tend to be located farther away than SNPs from the regulated genes (Fig. 2b).

Compared to eQTL analysis with SNPs, analysis with PAVs could improve the power for detecting more significant variations; for instance, many SNPs were found to be associated with the transcript expression of the gene Garb_10G032080 (Fig. 2c). This gene has been reported previously as a candidate gene that is known to bind ubiquitin and is associated with cotton fiber length [23]. The top signal of SNPs was located 28.4 kb downstream of the Garb_10G032080 gene (*P* = 1.24 ×  10^ −9^). We observed a PAV (NRS_166316), located 1.3 kb downstream of the Garb_10G032080 gene, which was more significant than the top SNP signal (*P* = 7.26 ×  10^ −15^). The gene expression level of Garb_10G032080 is significantly lower in the presence of NRS compared with that of its absence (Fig. 2d). These findings underscore the importance of considering structural variations in eQTL analyses; nevertheless, more functional gene studies should be carried out in the future.

### NRSs reduce comparative genomic bias

**Fig. 3:**
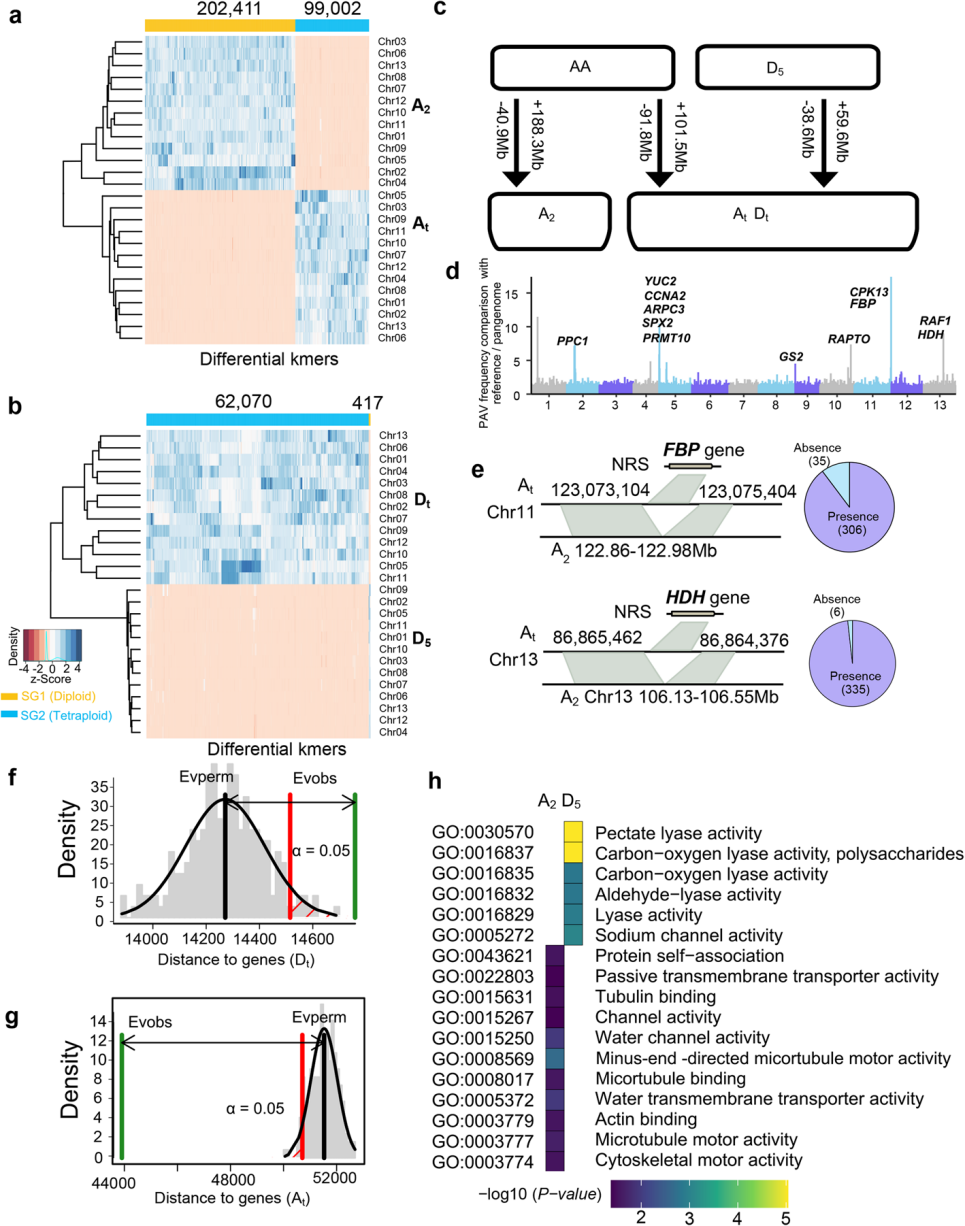
Sequence gain and loss after polyploidization. **a**, **b** Unsupervised hierarchical clustering was used to identify diploid and tetraploid genome-specific kmers. The heatmap indicates the *Z*-scaled relative abundance of genome-specific kmers, and the color bar on the top axis indicates the kmer assigned to a given genome. **c** Schematic illustrating the increase or decrease in PAV sequences in cotton. **d** Distribution of intraspecific PAV sequences in the A_t_ subgenome. Some genes are shown. These sequences are absent in the A_2_ reference genome but present in NRSs and tetraploid genome sequences. **e** The *FBP* and *HDH* variation in the A_2_ pangenome and A_t_ subgenome. The pie chart represents the gene presence frequency in the 341 accessions. **f**, **g** A permutation test was used to assess the distance of lost sequences from genes in the A_2_ and D_5_ genomes. The green bars represent the observed lost sequence with gene distances, and the black bars represent the relationship between random sequences and gene distance. **h** GO enrichment of genes in lost sequences in A_2_ and D_5_

A previous comparative genomic study revealed extensive PAV sequences between diploid and tetraploid cottons [12]. However, the origin of the PAV sequence after polyploidization is difficult to determine. For example, diploid-specific sequences cannot be distinguished from sequences lost in polyploids or nascent in diploids. We addressed the problem of the origin of PAVs through genome-specific kmers and pangenome comparisons (Additional File 1: Fig. S6). First, by comparing the reference genome sequences between A_2_ and A_t_, we found 202,411 and 99,002 genome-specific kmers, respectively (Fig. 3a), indicating that the A_2_ genome acquired a very large genome-specific kmers after speciation compared with A_t_. Similarly, we also found 417 and 62,070 genome-specific kmers in D_5_ and D_t_, respectively (Fig. 3b), and genome-specific kmers were rare in D_5_, suggesting that they might have been conserved after polyploidization. In addition, we found that 43,007 genome-specific kmers were shared between A_t_ and D_t_, implying that the genome-specific kmers in the two subgenomes coincided.

Second, we divided the A_2_ pangenome into 1 kb segments and aligned them to A_t_ to identify A_2_-specific sequences. The A_2_-specific sequences containing A_2_-specific kmers were considered nascent in A_2_ (see the “Methods” section). Otherwise, they represented lost sequences in the A_t_ subgenome. In total, we found that 280 Mb of sequences were specific to A_2_, of which 188.3 Mb were nascent in A_2_ and 91.8 Mb were lost in the A_t_ subgenome (Fig. 3c). For nascent sequences in the A_t_ subgenome, a pangenome comparison can reduce bias from reference genomes. We divided the A_t_-genome sequences and aligned them to the A_2_ reference genome to identify A_t_-specific sequences. This analysis gave rise to 201 Mb of sequences that were not matched. However, by aligning the A_t_-genome sequence to the A_2_ pangenome, the length of unaligned sequences was reduced to 142.4 Mb, of which 71.2% (101.5 Mb) contained A_t_-specific kmers that were considered nascent sequences in the A_t_ subgenome. Furthermore, although the nascent sequences in the A_t_ genome amounted to only 101.5 Mb, they comprised 60.9% of the full-length LTR retrotransposons in the A_t_ subgenome. We also compared them with the D_5_ genome to identify nascent sequences in D_t_. It was found that 59.6 Mb of nascent sequences contained 73.6% of full-length LTR retrotransposons in the D_t_ subgenome. In summary, we obtained the numbers of nascent and lost sequences in tetraploid cotton. The genome sizes of the two subgenomes were both expanded, but the A_t_ subgenome had fewer nascent sequences than the A_2_ genome (A_t_ ~ 101.5 Mb vs. A_2_ ~ 188.3 Mb), as well as more lost sequences (A_t_ ~ 91.8 Mb vs. A_2_ ~ 40.9 Mb), causing a decrease in the size of the A_t_ subgenome.

To test the accuracy of the use of genome-specific kmers for determining the source of PAV sequences, we checked the coverage of A_t_-specific kmers in a 1310 Mb A_t_ shared sequence and revealed that only 116.2 kb sequences contained the A_t_-specific kmer, which suggests the high accuracy of determining tetraploid nascent sequence by genome-specific kmers. Similarly, we examined the A_2_-specific kmers in 1341 Mb of A_2_ shared sequences and found that 224.6 Mb of the sequence contained the A2-specific kmers, implying possible underestimation of the content of nascent sequences in A_2_. Some nascent sequences may be highly similar to the A_t_ sequence, so they cannot be identified from genome comparison.

In addition, we found that ~ 60 Mb of the PAV sequences from the diploid A-genome comparison were intraspecific variations, which appeared in only a few accessions. The intraspecific PAVs were unevenly distributed across the genome (Fig. 3d). The longest PAV sequence was 124 kb and contained 21 genes (from 122.86 Mb to 122.98 Mb on chromosome 11). In Fig. 3e, two non-reference genes are highlighted as intraspecific variations. The *fructose-1,6-bisphosphatase* (*FBP*) gene plays a key role in sucrose synthesis and metabolism [24], participating in sugar regulation during cotton fiber development. We observed that *FBP* was lost on chromosome 11 of the “Shixiya 1” reference genome; nevertheless, this nonreference gene was present in 306 (89.7%) *G. arboreum* accessions. Similarly, *histidinol dehydrogenase* (*HDH*), associated with root growth [25], was missing on the A_2_ reference genome but present in 98.2% (335) of *G. arboreum* accessions. In total, 1324 nonreference genes were included in the intraspecific PAVs, which were considered to be lost in previous reference genome studies.

We also found that the sequence loss in tetraploid cotton was unbalanced between the two subgenomes. Analysis of the relationship between lost sequences and genes in diploids showed that the lost sequences were closer to genes than were random sequences in A_2,_ but the opposite pattern was observed for D_5_. Meanwhile, the missing sequences in D_5_ were farther away from the genes than those in A_2_ (Figs. 3f, g). Further analysis showed that the lost sequences in gene regions and their flanking 2 kb regions were related to 4088 and 2920 genes in A_2_ and D_5_, respectively. In the A_t_, lost genes were involved in multiple lyase activities, including pectate lyase activity, carbon–oxygen lyase activity, and aldehyde lyase activity (Fig. 3h), which might shape the adaptability of cotton to diverse environmental conditions. In the D_t_, lost genes were involved in protein self-association, tubulin binding, and channel activity, which were associated with cotton fiber properties and response to external stimuli.

### The evolutionary features of G. arboreum pangenes

**Fig. 4:**
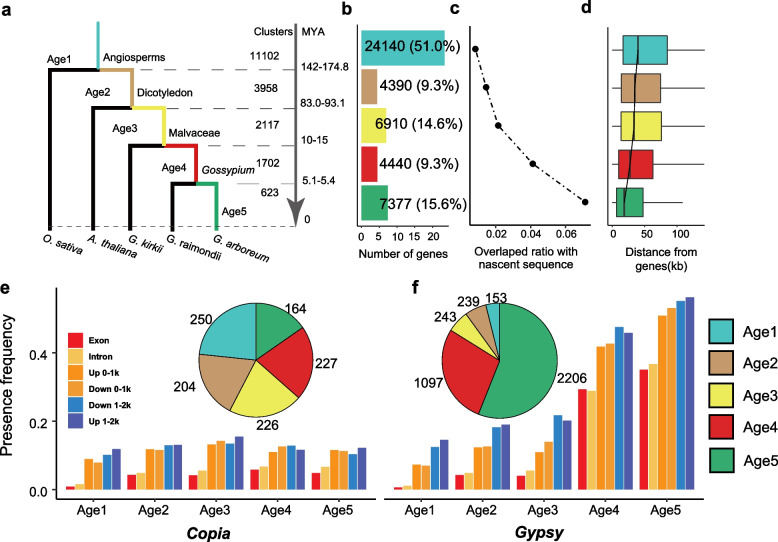
Gene age in the *Gossypium arboreum* pangenome. **a** Gene age distribution in the pangenome*.* The number indicates the number of gene clusters in each category. **b** The number of genes per gene catalog. **c** The proportion of nascent sequences overlapping with different ages of genes. **d** The boxplot shows the distance between nascent sequences and genes of different ages. **e** The *Gypsy* and *Copia* transposon insertions in *G. arboreum* pangenes near 2 kb regions. The pie charts represent the number of genes in which the coding regions contain LTR domains

According to the phylogenetic tree, we assigned the 47,257 pangenes to five age catalogs (Fig. 4a). The oldest gene group was orthologous to genes in the monocot plant *O. sativa*, which included 51% of the genes. A total of 15.6% of the youngest genes were only present in pangenes (Fig. 4b), which could be considered species-specific genes that diverged from *G. raimondii* approximately 5.1–5.4 MYA. After linking gene age to conservation, we found that older genes had a higher presence frequency in the population; in contrast, new genes had a higher evolution rate. The gene length, expression level, and exon number were correlated with gene age, with an increase in the median number from the youngest to the oldest group (Additional File 1: Fig. S7), consistent with previous findings in *O. sativa*, *A. thaliana*, and *H. sapiens* [26–29].

We explored the relationship between nascent sequences and gene age in the pangenome and found that species-specific genes were more likely to be near nascent sequences and thus likely to reside closer to LTR retrotransposons (Fig. 4c). A total of 7.1% of species-specific genes in Age5 overlapped with nascent sequences, but in Age1, only 0.8% of genes overlapped with nascent sequences. With increasing gene age, the average distance to the nascent sequence increased (Fig. 4d).

In tomato and Arabidopsis, *Copia* retrotransposons were found to be located preferentially within or near genes, in contrast to *Gypsy* retrotransposons [30]. However, we found that the *Gypsy* superfamily existed more frequently than *Copia* in *G. arboreum*, especially in younger (Age5) genes (Fig. 4e, f). This opposite trend may be related to the ultrahigh *Gypsy* content in *G. arboreum*. We found that the coding sequences of 6217 genes contained TE domains (Additional File 2: Table S4), of which 3938 (63.3%) were caused by *Gypsy* insertion and were mostly concentrated in species-specific genes (2206 genes). This observation indicates that *Gypsy* may play a key role in the evolution of new genes in *G. arboreum*.

### Gene loss and gain after polyploidization

**Fig. 5:**
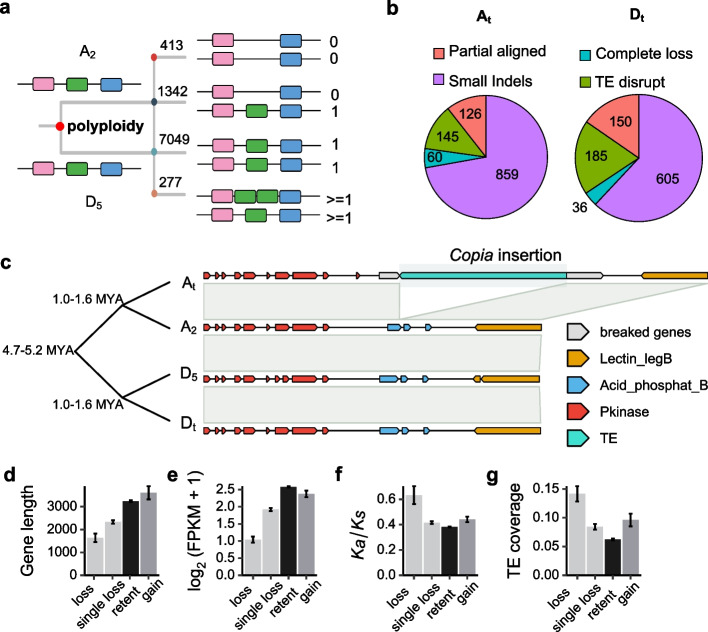
Single-copy gene states after polyploidization. **a** A proposed model of the status of single-copy genes after polyploidization. All genes belong to single copies in both diploid progenitor genomes, and the status 0 or 1 represents gene loss or retention in tetraploid subgenomes. **b** Pie charts showing single-copy gene loss in different situations. **c** The phylogenetic tree shows the relationship in the four genomes. The figure shows that the gene on chromosome A07 of the A_t_ subgenome was lost, accompanied by *Copia* LTR retrotransposon insertion. This gene was present in the other three genomes. The colored boxes indicate coding regions and protein domain information from the Pfam database. **d**–**g** Gene characterization with different status. Mean and SE values are indicated

We explored single-copy gene variation patterns in both diploid (A_2_ and D_5_) and tetraploid (AD_1_) cotton. We divided the 9081 single-copy genes from the A-clade pangenes and D_5_ genes into four groups (Fig. 5a). The first group (two copies completely lost in the tetraploid) contained 413 genes, and 72.1% of lost genes were only present in the *Gossypium* genus at age 4 (Additional File 1: Fig. S8). The second group had genes reverted to a single copy from two copies, of which 1342 were lost with certain functional implications. Genes involved in DNA repair and targeted to the organelles tended to have a single copy after polyploidization. In addition, the frequency of gene loss was not equal between subgenomes, with higher rates of gene loss in the A_t_ subgenome (777 genes in A_t_ and 565 genes in D_t_), consistent with findings in previous studies [31]. In the third group (balance), both copies were retained in the tetraploid, involving 7049 genes. The last group (277 genes) showed the lowest proportion, including the single-copy genes with copy numbers that increased after polyploidization.

To investigate the effect of TE activity on gene copy number loss, we analyzed TE insertions in gene body regions. We found that 145 (12.2%) and 185 (18.9%) single-copy genes were lost in A_t_ and D_t_ following LTR retrotransposon insertion, respectively (Fig. 5b), suggesting that TEs played an important role in single-copy gene loss, especially for D_t_. An example of gene loss in tetraploid associated with *Copia* insertion is shown in Fig. 5c. The gene Garb_07G024840 (located in the A_t_ genome from 5.54 Mb to 5.78 Mb on chromosome A07) has a legume lectin domain related to plant defense against predators and was lost in the A_t_ genome but retained in the other three genomes (Fig. 5c). We examined the gene features of single-copy genes in different states. Genes with copy number loss had shorter coding length, lower expression levels, and higher *K*_*a*_*/K*_*s*_ values and TE coverage (*P* < 2.2 ×  10^ −6^, Wilcox test). The genes with copy number gain were more conserved than those with copy number loss (Fig. 5d–f).

### TE drives genome size variation after polyploidization

**Fig. 6:**
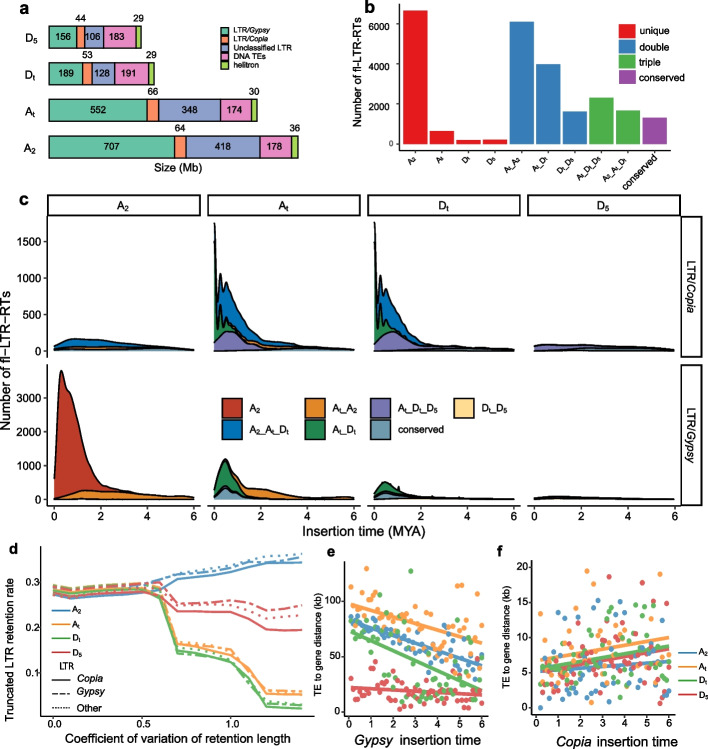
LTR retrotransposon dynamics after polyploidization. **a** TE components in diploid and tetraploid cotton. **b** Comparison of LTR retrotransposon abundance in a different cluster. “A_2_” only has members in the A_2_ genome. “A_t__D_t_” represents only the tetraploid genome, and “common” has members in all four genomes. **c** Amplification time of different classification clusters. **d** The LTR retrotransposon retention rate in four genomes. The *X*-axis indicates the CV (coefficient of variation) threshold of the truncated LTR length in four genomes, and the right indicates the LTR retention length with high variability in the homologous regions. **e**, **f** Linear regression analysis of insertion distances and insertion times for *Gypsy* and *Copia*

The most striking genome feature of polyploid cotton is the distinction of genome size compared with that of diploid progenitors. The D_t_ subgenome expanded from 750 Mb of D_5_ to 796 Mb, and the A_t_ subgenome (1437 Mb) was significantly reduced compared with A_2_ (1620 Mb). Comparison of genome components showed that *Gypsy* retrotransposons predominantly contributed to expansion of the A_2_ genome. The major types of TEs in D_t_ were more abundant than those in D_5_ (Fig. 6a), which might be responsible for the genome size evolution between the A and D genomes. The majority of fl-LTR (full-length LTR) retrotransposon copies from ancient bursts were usually truncated. However, some truncated fl-LTRs might have an intact structure in other *Gossypium* genomes, suggesting that the pangenome can help identify more full-length LTRs (see the “Methods” section). By using the four genome sequences, we constructed a pan-TE library and identified 13,865, 9991, 5661, and 2841 fl-LTR retrotransposons in the A_2_, A_t_, D_t_, and D_5_ reference genomes, respectively.

The analysis of fl-LTR retrotransposon insertion time showed that *Gypsy* elements underwent a burst in the A genome after speciation divergence (~ 0.6 MYA). *Copia* elements were relatively conserved among species, except for a small expansion in A_t_ and D_t_ (Additional File 1: Fig. S9). To elucidate the effect of polyploidization on LTR retrotransposon activity, we categorized the LTRs in the four genomes into different clusters based on sequence identity (Fig. 6b). We found extensive genome-specific LTR retrotransposons amplified in the A_2_ genome and only a few clusters with low copy numbers amplified in the other three genomes. The A_2__A_t_ cluster had more copy numbers than D_5__D_t_, consistent with the fact that the A genome was larger than the D genome. Interestingly, the A_t__D_t_ shared cluster was massively amplified, representing concerted proliferation in tetraploids. The A_2__A_t__D_t_ cluster included the LTR retrotransposon that might be transferred from A_t_ to D_t_. These results suggested that LTR retrotransposons were subject to exchange between the two subgenomes in tetraploid cotton. The LTR retrotransposons conserved in all four genomes belonged to the oldest copies that might have already existed before polyploidization.

We calculated the burst time of different clusters (Fig. 6c). The LTR retrotransposon specific to either the A or D genome amplified approximately 2 MYA, consistent with the time of allopolyploidization, suggesting that the two subgenomes were subject to independent evolution in the diploid lineages. Notably, in the *Gypsy* superfamily of the tetraploid-specific cluster, A_t__D_t_ dominated the new sequence after polyploidy, and LTR retrotransposon amplification was coincident between the two subgenomes (Additional File 1: Fig. S10).

We clustered the reverse transcriptase (RT) domains from the fl-LTR retrotransposon and found parallel subgenome evolution of copy number variation in different families (Additional File 2: Table S5). For example, the *Tekay* family was abundant in the cotton clade with the A genome, but the copy number increased significantly in D_t_ compared with D_5_ and decreased in A_t_ compared with A_2_. This was similar to a previous finding of CRG (Centromere Retroelement *Gossypium*), which was detected in the centromere regions of D_5_, A_t_ and D_t_, while none existed in all diploid A-clade species, indicating that CRG in D_t_ might invade the centromere regions of the A_t_ subgenome [32].

The TE content was affected by both amplification and elimination. Accordingly, rapid LTR retrotransposon elimination can reduce TE diversity and decrease genome size. We analyzed the consequences of TE elimination events in each genome. The 32,059 homologous truncated LTR retrotransposon regions were used to analyze the retention rates in the four genomes. Most of the homologous regions had a similar retention rate in the four genomes. However, we found that in some homologous regions with highly variable retention lengths, the average retention rate in tetraploid cotton was lower than that in diploid cotton (Fig. 6d), and that in D_t_ was slightly lower than that in the At subgenome, which suggests a faster LTR retrotransposon elimination rate in tetraploid cotton. This was consistent with the above genome PAV results (Fig. 2c). In addition, we found no difference in the LTR retrotransposon retention rate across different TE superfamilies in the whole genome (*P* > 0.05, *t* test), which suggests that the key factor affecting the LTR retrotransposon component is insertions and that the deletion rate is stable in the cotton genome.

In terms of the relationship between LTR retrotransposons and genes, we explored the relationship between burst time and insertion distances in four genomes (Fig. 6e, f). For the *Gypsy* superfamily, the recent insertion was predominantly located far from genes, and the magnitudes of the slopes of the regression lines were −6.82, −5.92, − 9.02, and −1.12 for A_2_, A_t_, D_t_, and D_5_, respectively. However, *Copia* insertions tended to occur around genes, and the magnitudes of the slopes were positive at 1.24, 1.73, 0.669, and 0.554, respectively. This suggested that these two LTR retrotransposon families have different effects on the genome.

Despite the LTR retrotransposon burst after polyploidization, the distance of neighboring homologous genes tended to be conserved, and the ratio of diploid and tetraploid homologous distances had a strong peak at 1 (0 on a log scale) (Additional File 1: Fig. S11). These results indicated that the cotton genome might have responded to selection to regulate the relative positional stabilization of homologous genes.

## Discussion

Previous studies on crop pangenomes have found that a single reference genome is insufficient for studying genetic diversity. Thus, we constructed the pangenome of the diploid A-genome cotton to uncover the genome variation effect of polyploidization in cotton. The diploid cotton A-clade pangenome was ~ 2.1 Gb, with 47,257 genes, of which ~ 87% were core genes, indicating a higher core gene content than observed in tomato (74.2%), *Arabidopsis thaliana* (70%), *Brassica napus* (62%), and bread wheat (64%) but one similar to that in tetraploid cotton (84.8%) [16, 21, 33, 34]. We observed that 11.9% of the pangenes exhibited PAVs, and the cultivation adaptations of diploid cotton have resulted in a series of dispensable genes in different accessions, indicating the diverse genetic makeup of cotton from different geographic origins. The pangenome also provides a valuable resource for eQTL studies, as it offers a broader spectrum of genetic variation than a single reference genome. With a diverse set of accessions included in the pangenome, it becomes possible to identify eQTLs that are specific to particular genotypes or populations, capturing the breadth of genetic diversity present in cotton. This information can be harnessed to develop a more comprehensive understanding of the genetic basis of complex traits and their regulation. However, it is noteworthy that the selection of accessions has been predominantly biased towards the CHN group. Therefore, future research efforts should prioritize expanding the geographical representation of accessions, which is likely to enrich our comprehensive understanding of the genetic diversity within *G. arboreum*.

Previous studies have shown that polyploid formation is accompanied by extensive sequence insertions and deletions in Arabidopsis, tobacco, wheat, and rapeseed [35–37]. These SVs can drive important phenotypic variation, but the presence of SVs resulting from intraspecific variation will lead to overestimates of the changes that occurred after polyploidization. Pangenome analysis can distinguish between diploid and tetraploid sequence differences after polyploidy. Here, our comparative pangenome approach provides a deeper understanding of the genome structure change underlying polyploid evolution. The numbers of SVs following polyploidization were corrected and revealed that both subgenomes in polyploid cotton underwent expansion. These results demonstrate that the pangenome can provide a full view of the mechanisms of SV formation, which can help explain the complex structure of genome evolution.

Species-specific genes may be an important and continuous gene pool for understanding gene evolution, which can lead to a new adaptive mechanism and phenotype. Resolving the role of transposons in the formation of species-specific genes in the cotton genome is necessary, and the abundance of dispensable genes in the pangenome provides a rich resource for studying species-specific genes. Our results confirm that *Gypsy* superfamily LTR retrotransposons are used for species-specific gene innovation.

Differences from ancestral progenitors lead to subgenome dominance, and polyploidy reconciles conflict, although through multiple mechanisms [38]. A previous study revealed that subgenome differences in TE density probably underlie subgenome dominance [39]. We constructed a pan-TE dataset in tetraploid and progenitor species to facilitate a comparison of TE composition across species. The LTR retrotransposon clustering indicated that the two subgenomes were becoming more similar, and the parallel evolution of the two subgenomes in cotton will result in similar subgenome LTR retrotransposon compositions.

## Conclusions

The diploid A-genome cotton pangenome has illuminated the intricate genetic diversity and evolutionary dynamics of TEs underlying polyploidization in cotton. This investigation underscores the importance of pangenome analysis in deciphering the role of TEs in driving genome evolution.

## Methods

### Pangenome construction

Genome sequencing data of 344 accessions were collected from previous studies [12, 23, 40] (Additional File 2: Table S1). Raw Illumina reads were filtered using fastp with default parameters [41]. Consistent with a previous study in tomato [21], high-quality clean reads from each sample were de novo assembled by the MEGAHIT assembler [42]. Contigs longer than 500 bp were retained and used to align to the “Shixiya 1” reference genomes with nucmer [8, 43]. Contig alignments with continuous alignment were defined as those longer than 300 bp and with a sequence identity higher than 90% (-I 90 -l 300 -q). If the aligned contigs also contained continuously unaligned regions longer than 500 bp, the unaligned regions were extracted as unaligned sequences. The unaligned contigs and unaligned sequences were searched against the nt database [44], and only the contigs matching the Viridiplantae sequences were retained. The clean nonreference sequences (NRSs) were merged and subjected to the removal of redundant sequences using CD-HIT with default parameters [45]. To further remove redundant sequences, rmRedundant from EUPAN was used to cluster sequences and extract representative sequences [46]. The final nonredundant sequences were aligned to the reference genome using blastn to ensure that there was no duplication with the reference genome. The final NRSs and the “Shixiya 1” reference sequence were combined into the pangenome.

### Prediction of genes in NRSs

RepeatModeler was used to construct the custom repeat library for the pangenome [47]. The gene model annotations were obtained from three rounds of training with the MAKER2 pipeline [48]. RNA-Seq data from previous reports were mapped to the pangenome using HISAT2 [12, 49] and de novo assembled using Trinity [50]. Cotton expressed sequence tags (ESTs, May 2019) were downloaded from the NCBI database. Protein sequences of cotton were downloaded from the NCBI and UniProtKB databases. These data were used for gene prediction in the first round, and then the gene annotation files were collected to train SNAP [51]. In the second round, the trained SNAP model and predicted gene models were used as input. For the third round, we retrained SNAP and ran MAKER again. For filtering of nonreference genes, genes with fewer than 50 bp and overlapping with more than 50% repeat sequences were excluded.

Genes were annotated by submitting the protein sequences to the eggNOG online website [52]. GO enrichment analysis of dispensable genes was performed by the R package clusterProfiler [53].

### Analysis of gene presence/absence variations (PAVs)

The raw Illumina genome sequence reads were aligned to the pangenome using BWA-MEM with default parameters [54]. Then, SGSGeneLoss was used to determine gene presence or absence [55] (minCov = 2, lostCutoff = 0.2). If more than 80% of exon regions were covered by short reads, this gene was considered present; otherwise, it was considered absent.

Based on the binary PAV data of dispensable genes, the maximum-likelihood phylogenetic tree was constructed by IQ-TREE with 1000 bootstrap replicates [56]. The tree file was visualized using the R package ggtree. We utilized a customized Python script to perform a thorough analysis of pan-genome and core genome sizes across 1000 random samplings, spanning pairs to combinations of 340 genomes. Each iteration involved the random selection of gene combinations, and the quantities of pan-genes and core genes were calculated. *K*_*a*_/*K*_*s*_ values between pangenes and orthologous genes were calculated by TBtools [57]*.*

### eQTL detection

Sequence PAVs in the pangenome were called by pangenome construction and a downstream pangenome analysis pipeline (PSVCP) [58], and then the PAV genotype and SNP data were merged to perform eQTL analysis. The RNA-seq data of 216 accessions with gene expression data were obtained from our previous study [23]. We conducted *cis*-eQTL mapping of the five fiber developmental stages by tensorQTL [59]. For details of the eQTL analysis, please refer to our previous research [23].

### Determination of the origin of the tetraploid genome sequence

We aligned the A_t_ subgenome sequence of AD_1_ with the A_2_ reference genome to identify the sequence in tetraploids that emerged after polyploidization or intraspecific differentiation. The methods were as follows: (I) the tetraploid genome was split into 1 kb fragments after removing N bases. (II) The obtained fragments were mapped against the diploid assembly using BWA-MEM [54]. (III) Alignment was processed to filter out small duplicates and identify mapping coordinates. (IV) We extracted the unmapped fragments and merged adjacent fragments into a single sequence. The PAV sequences excluding intraspecific variation were obtained from the unmapped sequence of the reference genome alignment after subtracting the pangenome alignment. Mosdepth [60] was used to check the sequencing coverage in genomic intervals. These steps were performed separately for the two genome pairs (A and D).

The SubPhaser software was used to identify genome-specific kmers [61]. (1) Jellyfish was used to scan and count 15-mers in the genome [62], with an extraction of kmers exceeding a count of 100 for further analysis. (2) For each homoeologous chromosome set, the relative abundance of k-mers in one genome was more than twice that of another genome. (3) After normalizing k-mer matrices, the k-means algorithm was used to cluster kmers into N groups. We performed 1000 bootstrap resamplings of these k-mers (each 50%) for statistical inference.

To identify the PAV fragments lost after polyploidization in diploids, we masked the A_2_-specific kmers in each PAV sequence using BBDuk package from BBMap and then connected the mask regions with spacing of less than 50 bp by BedTools [63, 64]. When masked fragments accounted for less than 20% of PAV sequences, these sequences were considered to be lost after polyploidization. To compare the genomic features of the lost sequences, random sampling of the genomic fragments was used as a control with the R package regioneR [65]. Lost sequences that covered 2 kb upstream and downstream flanking regions of genes were defined using the intersect function in BEDTools and evaluated for differences using the t.test function in R. GO enrichment was performed using the R package ClusterProfiler.

### Estimation of the age of pangenes

OrthoFinder was used for phylogenetic clustering of *Gossypium raimondii, Gossypium kirkii*, *Oryza sativa*, *Arabidopsis thaliana*, and A-clade pangenes [66, 67]. The A-clade pangenes were allocated into five age bins: From the youngest to oldest, (I) only present in the A-clade pangenome, (II) shared among cotton species, (III) shared among Malvaceae, (IV) shared with dicotyledonous *A. thaliana*, and (V) shared with monocotyledonous *O. sativa*. TEsorter was used to examine the transposon domain of each pangene [68].

### Single-copy gene state analysis

The protein clustering results were calculated by a previous method for gene age to examine the status of single-copy genes in tetraploids. For each gene family, if the member number ratio was 1:1:2, 1:1:0, 1:1:1, or 1:1:*n* (*n* > 2) among A_2_, D_5_, and (AD)_1_, we considered it as retained, lost, reverted to a single copy, or gained, respectively.

Within each gene family, we performed a pairwise comparison of gene structures (gene length, exon number, K_a_/K_s_, expression level, and TE coverage). First, GXF Stat in TBtools was used to generate the gff file of statistics. Then, customized Python scripts were used to calculate the above genome features.

Genes disrupted by TEs were traced between tetraploid and two diploids. We used BEDTools to extract the gene sequences lost in diploids and align them to their tetraploid counterparts by BLASTN (-evalue 1e-6 -max_target_seqs 5). If the single aligned length exceeded 90% of the query gene length, it was considered a gene annotation omission or gene loss driven by small variations. To identify genes that have been disrupted by the insertion of transposable elements (TEs), we aligned the diploid gene sequences to the tetraploid genome and looked for multiple alignments within a 25-kb distance and TE annotations in the gap regions of the alignments.

### Repeat annotation and LTR retrotransposon clustering

To generate comparable repeat annotations, we used the EDTA pan-TE pipeline to generate TE annotations from the genome sequences of A_2_, A_t_, D_t_, and D_5_, as described in a maize pangenome study [69, 70]. To construct the pan-TE library, we used the following pipeline. First, EDTA software was used to generate the raw TE library for the genome. Second, multiple TE libraries were aggregated into one file, and the redundant sequences were removed. Third, misidentified TEs were removed to obtain the complete TE library. Finally, the pan-TE library was used to remask all genomes.

LTR retrotransposons were extracted for downstream analysis. To calculate the age of the full-length LTR retrotransposons, divergence (K) was estimated based on the divergence between two LTR copies. The Jukes–Cantor model [71] was applied for correction, and then the ages of each copy were estimated using *T* = *K*/*r*, in which *r* is the nucleotide mutation rate (*r* = 9 × 10^ −9^) [72].

Similar to a previously reported method in wheat [73], vmatch [74] was used to cluster the full-length LTR sequences with 90/90 cutoffs: -dbcluster 90 90 -exdrop 5 -identity 90 -d -seed length 15. The genome specificity of each cluster was determined with the following decision tree: (1) if a cluster had more than 90% of the members from the single genome, it was defined as a genome-specific cluster. (2) The cluster members from the two genomes contributed more than 90%. (3) Cluster members from one subgenome accounted for < 10%. (4) The remaining cluster was considered a conserved cluster in cotton. The amplification lifespan for each cluster was defined as ranging from the 5th to 95th percentile, corresponding to the oldest to the youngest insertion.

### Analysis of deletion rate of LTR retrotransposon

To investigate the LTR retrotransposon deletion rates, the retention length of truncated LTR retrotransposons in homologous regions was examined. The steps were as follows: (1) only the full-length LTR retrotransposons were used to mask genomes to identify genomic regions containing LTR retrotransposon sequences by RepeatMasker [75]. (2) To identify homologous regions, other genome sequences were aligned to the A2 pangenome with nucmer from the MUMMER package (-g 500). The nucmer outputs were filtered with a delta filter (-g) and converted to aligned coordinates with show-coords (-r -I 70 -T -H). (3) A custom Python script was used to identify common homologous regions between A_2_, A_t_, D_t_, and D_5_ using the “show-coords” results as input. (4) After searching the LTR retrotransposon annotations in each homologous region of the four genomes, the LTR retrotransposons existing in only one alignment in each of the four genome homologous regions were retained for further analysis. (5) Multiple sequence alignments for each multifasta file were performed by using MAFFT [76] (-op 5 -ep 0). (6) The remaining LTR retrotransposon length was calculated for each genome and compared to the total length of the multiple sequence alignment results. The above results were used to compute the deletion rate of LTR retrotransposons in each homologous region. Because of a few retention length variations in all homologous regions, different cutoffs of the coefficient of variation (CV) were used for analysis of the LTR retention length of each selected locus.

### Analysis of distance between LTR retrotransposons and genes

The distanceToNearest function from the R package GenomicTuples was used to calculate the distance from each full-length (fl-LTR) retrotransposon to a nearby gene. The R package regioneR [65] was used to calculate the insertion distances of different types of LTR retrotransposons in genes by randomly sampling 1000 times from the genome with the same fl-LTR retrotransposon distribution. A permutation test was performed to assess the distance between the LTR retrotransposons and genes compared to that with random sequences.

To compare the distance between adjacent homologous genes, we only considered homologous genes existing in four genomes with restricted directions. Then, we calculated the spacing of each adjacent homologous gene. For randomization, we preserved the gene orders but randomized the distances between genes. A chi-square test was used to calculate the significance of the actual and random gene positions.

### Supplementary Information

**Additional file 1. Fig. S1.** BUSCO statistics and NRSs alignment distribution. **Fig. S2.** Distribution of different classifications of genes in the reference genome and non-reference sequences. **Fig. S3.** The PCR experiment confirmed the five non-reference genes. **Fig. S4.** Gene feature comparisons between core, softcore, shell, and cloud genes. **Fig. S5.** GO enrichment for selected and unselected genes. **Fig. S6.** Schematic diagram illustrating the overall process of identifying the A2-nascent sequences and At-lost sequences. **Fig. S7.** Comparison of gene characteristics with different ages. **Fig. S8.** Gene age distribution in different single-copy gene status. **Fig. S9.** The LTR-RT amplification patterns between four genomes. **Fig. S10.** The lifespan of the LTR-RT cluster. **Fig. S11.** The adjacent homologous genes distance between diploid and tetraploid cotton.

**Additional file 2. Table S1.** Summary of whole genome sequencing data. **Table S2.** NRS statistics. **Table S3.** PCR primer used in the validation of five selected non-reference genes. **Table S4.** Summary of CDS regions that contain TE domains.**Table S5.** LTR-RT family content in four genomes.

## Data Availability

All genome and transcriptome sequencing data used in this article are included in supplementary information files. The cotton pangenome data and non-reference sequence gene annotations, and custom code are available at public repositories (10.6084/m9.figshare.24354895 [77].

## Abbreviations

BUSCO: Benchmarking Universal Single-Copy Orthologs
CV: Coefficient of variation
eQTL: Expression quantitative trait loci
GO: Gene Ontology
SV: Structural variation
LTR-RT: Long terminal repeat retrotransposons
PAV: Presence/absence variation
PCR: Polymerase chain reaction
TEs: Transposable elements
TWAS: Transcriptome-wide association study

## References

[1] Wendel JF (1989). New World tetraploid cottons contain Old World cytoplasm. Proc Natl Acad Sci U S A.

[2] Senchina DS, Alvarez I, Cronn RC, Liu B, Rong J, Noyes RD (2003). Rate variation among nuclear genes and the age of polyploidy in *Gossypium*. Mol Bio Evol.

[3] Wang M, Wang P, Tu L, Zhu S, Zhang L, Li Z (2016). Multi-omics maps of cotton fibre reveal epigenetic basis for staged single-cell differentiation. Nucleic Acids Res.

[4] Song Q, Zhang T, Stelly DM, Chen ZJ (2017). Epigenomic and functional analyses reveal roles of epialleles in the loss of photoperiod sensitivity during domestication of allotetraploid cottons. Genome Biol.

[5] Conover JL, Wendel JF (2022). Deleterious mutations accumulate faster in allopolyploid than diploid cotton (Gossypium) and unequally between subgenomes. Mol Biol Evol.

[6] You J, Lin M, Liu Z, Pei L, Long Y, Tu L (2022). Comparative genomic analyses reveal cis-regulatory divergence after polyploidization in cotton. Crop J.

[7] Wang M, Tu L, Yuan D, Zhu D, Shen C, Li J (2019). Reference genome sequences of two cultivated allotetraploid cottons, *Gossypium hirsutum* and *Gossypium barbadense*. Nat Genet.

[8] Wang M, Li J, Wang P, Liu F, Liu Z, Zhao G (2021). Comparative genome analyses highlight transposon-mediated genome expansion and the evolutionary architecture of 3D genomic folding in cotton. Mol Biol Evol.

[9] Wang K, Huang G, Zhu Y (2016). Transposable elements play an important role during cotton genome evolution and fiber cell development. Sci China Life Sci.

[10] Modzelewski AJ, Shao W, Chen J, Lee A, Qi X, Noon M (2021). A mouse-specific retrotransposon drives a conserved Cdk2ap1 isoform essential for development. Cell..

[11] Yang Y, Wen X, Wu Z, Wang K, Zhu Y (2023). Large-scale long terminal repeat insertions produced a significant set of novel transcripts in cotton. Sci China Life Sci.

[12] Huang G, Wu Z, Percy RG, Bai M, Li Y, Frelichowski JE (2020). Genome sequence of *Gossypium herbaceum* and genome updates of *Gossypium arboreum* and *Gossypium hirsutum* provide insights into cotton A-genome evolution. Nat Genet.

[13] Yang X, Lee W-P, Ye K, Lee C (2019). One reference genome is not enough. Genome Biol.

[14] Montenegro JD, Golicz AA, Bayer PE, Hurgobin B, Lee H, Chan CKK (2017). The pangenome of hexaploid bread wheat. Plant J.

[15] Wang W, Mauleon R, Hu Z, Chebotarov D, Tai S, Wu Z (2018). Genomic variation in 3,010 diverse accessions of Asian cultivated rice. Nature.

[16] Li J, Yuan D, Wang P, Wang Q, Sun M, Liu Z (2021). Cotton pan-genome retrieves the lost sequences and genes during domestication and selection. Genome Biol.

[17] Varshney RK, Roorkiwal M, Sun S, Bajaj P, Chitikineni A, Thudi M (2021). A chickpea genetic variation map based on the sequencing of 3,366 genomes. Nature.

[18] Gordon SP, Contreras-Moreira B, Levy JJ, Djamei A, Czedik-Eysenberg A, Tartaglio VS, Session A (2020). Gradual polyploid genome evolution revealed by pan-genomic analysis of *Brachypodium hybridum* and its diploid progenitors. Nat Commun.

[19] Zhuang Y, Wang X, Li X, Hu J, Fan L, Landis JB (2022). Phylogenomics of the genus Glycine sheds light on polyploid evolution and life-strategy transition. Nat Plants.

[20] Bayer PE, Scheben A, Golicz AA, Yuan Y, Faure S, Lee H (2021). Modelling of gene loss propensity in the pangenomes of three Brassica species suggests different mechanisms between polyploids and diploids. Plant Biotechnol J.

[21] Gao L, Gonda I, Sun H, Ma Q, Bao K, Tieman DM (2019). The tomato pan-genome uncovers new genes and a rare allele regulating fruit flavor. Nat Genet.

[22] Vialle RA, de Paiva LK, Bennett DA, Crary JF, Raj T (2022). Integrating whole-genome sequencing with multi-omic data reveals the impact of structural variants on gene regulation in the human brain. Nat Neurosci.

[23] Wang M, Li J, Qi Z, Long Y, Pei L, Huang X (2022). Genomic innovation and regulatory rewiring during evolution of the cotton genus *Gossypium*. Nat Genet.

[24] Gě Q, Cūi Y, Lǐ J, Gōng J, Lú Q, Lǐ P (2020). Disequilibrium evolution of the Fructose-1,6-bisphosphatase gene family leads to their functional biodiversity in *Gossypium* species. BMC Genomics.

[25] Wang Z-A, Li Q, Ge X-Y, Yang C-L, Luo X-L, Zhang A-H, Xiao J-L, Tian Y-C, Xia G-X, Chen X-Y (2015). The mitochondrial malate dehydrogenase 1 gene GhmMDH1 is involved in plant and root growth under phosphorus deficiency conditions in cotton. Sci Rep.

[26] Guo Y-L (2013). Gene family evolution in green plants with emphasis on the origination and evolution of Arabidopsis thaliana genes. Plant J.

[27] Arendsee ZW, Li L, Wurtele ES (2014). Coming of age: orphan genes in plants. Trends Plant Sci.

[28] Stein JC, Yu Y, Copetti D, Zwickl DJ, Zhang L, Zhang C (2018). Genomes of 13 domesticated and wild rice relatives highlight genetic conservation, turnover and innovation across the genus Oryza. Nat Genet.

[29] Yin H, Li M, Xia L, He C, Zhang Z (2018). Computational determination of gene age and characterization of evolutionary dynamics in human. Brief Bioinform.

[30] Domínguez M, Dugas E, Benchouaia M, Leduque B, Jiménez-Gómez JM, Colot V, Quadrana L (2020). The impact of transposable elements on tomato diversity. Nat Commun.

[31] Fang L, Zhang Z, Zhao T, Zhou N, Mei H, Huang X (2022). Retrieving a disrupted gene encoding phospholipase A for fibre enhancement in allotetraploid cultivated cotton. Plant Biotechnol J.

[32] Luo S, Mach J, Abramson B, Ramirez R, Schurr R, Barone P (2012). The cotton centromere contains a Ty3-gypsy-like LTR retroelement. PLoS ONE.

[33] Gordon SP, Contreras-Moreira B, Woods DP, Des Marais DL, Burgess D, Shu S (2017). Extensive gene content variation in the Brachypodium distachyon pan-genome correlates with population structure. Nat Commun.

[34] Song J-M, Liu D-X, Xie W-Z, Yang Z, Guo L, Liu K (2021). BnPIR: *Brassica napus* pan-genome information resource for 1689 accessions. Plant Biotechnol J.

[35] Ramsey J, Schemske DW (2002). Neopolyploidy in flowering plants. Annu Rev Ecol Syst.

[36] Osborn TC, Chris Pires J, Birchler JA, Auger DL, Jeffery Chen Z, Lee H-S (2003). Understanding mechanisms of novel gene expression in polyploids. Trends Genet.

[37] Jiao Y, Wickett NJ, Ayyampalayam S, Chanderbali AS, Landherr L, Ralph PE (2011). Ancestral polyploidy in seed plants and angiosperms. Nature.

[38] Bird KA, VanBuren R, Puzey JR, Edger PP (2018). The causes and consequences of subgenome dominance in hybrids and recent polyploids. New Phytol.

[39] Freeling M, Woodhouse MR, Subramaniam S, Turco G, Lisch D, Schnable JC (2012). Fractionation mutagenesis and similar consequences of mechanisms removing dispensable or less-expressed DNA in plants. Curr Opin Plant Biol.

[40] Grover CE, Arick MA, II, Thrash A, Sharbrough J, Hu G, Yuan D, et al. Dual domestication, diversity, and differential introgression in Old World cotton diploids. Genome Biol Evol*.* 2022, 14(12). 10.1093/gbe/evac170 PMC9792962 36510772

[41] Chen S, Zhou Y, Chen Y, Gu J (2018). fastp: an ultra-fast all-in-one FASTQ preprocessor. Bioinformatics.

[42] Li D, Liu CM, Luo R, Sadakane K, Lam TW (2015). MEGAHIT: an ultra-fast single-node solution for large and complex metagenomics assembly via succinct de Bruijn graph. Bioinformatics.

[43] Marçais G, Delcher AL, Phillippy AM, Coston R, Salzberg SL, Zimin A (2018). MUMmer4: a fast and versatile genome alignment system. PLOS Computl Biol.

[44] Sayers EW, Bolton EE, Brister JR, Canese K, Chan J, Comeau Donald C (2021). Database resources of the national center for biotechnology information. Nucleic Acids Res.

[45] Li W, Godzik A (2006). Cd-hit: a fast program for clustering and comparing large sets of protein or nucleotide sequences. Bioinformatics.

[46] Hu Z, Sun C, Lu KC, Chu X, Zhao Y, Lu J, Shi J, Wei C (2017). EUPAN enables pan-genome studies of a large number of eukaryotic genomes. Bioinformatics.

[47] Flynn JM, Hubley R, Goubert C, Rosen J, Clark AG, Feschotte C (2020). RepeatModeler2 for automated genomic discovery of transposable element families. Proc Natl Acad Sci U S A.

[48] Holt C, Yandell M (2011). MAKER2: an annotation pipeline and genome-database management tool for second-generation genome projects. BMC Bioinformatics.

[49] Kim D, Paggi JM, Park C, Bennett C, Salzberg SL (2019). Graph-based genome alignment and genotyping with HISAT2 and HISAT-genotype. Nat Biotechnol.

[50] Grabherr MG, Haas BJ, Yassour M, Levin JZ, Thompson DA, Amit I (2011). Full-length transcriptome assembly from RNA-Seq data without a reference genome. Nat Biotechnol.

[51] Korf I (2004). Gene finding in novel genomes. BMC Bioinformatics.

[52] Cantalapiedra CP, Hernandez-Plaza A, Letunic I, Bork P, Huerta-Cepas J (2021). eggNOG-mapper v2: functional annotation, orthology assignments, and domain prediction at the metagenomic scale. Mol Biol Evol.

[53] Wu T, Hu E, Xu S, Chen M, Guo P, Dai Z, Feng T, et al. clusterProfiler 4.0: a universal enrichment tool for interpreting omics data. The Innovation*.* 2021, 2(3). 10.1016/j.xinn.2021.100141 PMC8454663 34557778

[54] Li H: Aligning sequence reads, clone sequences and assembly contigs with BWA-MEM. arXiv:1303.3997, 2013.

[55] Golicz AA, Martinez PA, Zander M, Patel DA, Van De Wouw AP (2015). Gene loss in the fungal canola pathogen Leptosphaeria maculans. Funct Integr Genomics.

[56] Minh BQ, Schmidt HA, Chernomor O, Schrempf D, Woodhams MD, von Haeseler A (2020). IQ-TREE 2: new models and efficient methods for phylogenetic inference in the genomic era. Mol Biol Evol.

[57] Chen C, Chen H, Zhang Y, Thomas HR, Frank MH, He Y (2020). TBtools: an integrative toolkit developed for interactive analyses of big biological data. Mol Plant.

[58] Wang J, Yang W, Zhang S, Hu H, Yuan Y, Dong J, Chen L (2023). A pangenome analysis pipeline provides insights into functional gene identification in rice. Genome Biol.

[59] Taylor-Weiner A, Aguet F, Haradhvala NJ, Gosai S, Anand S, Kim J, Ardlie K (2019). Scaling computational genomics to millions of individuals with GPUs. Genome Biol.

[60] Pedersen BS, Quinlan AR (2017). Mosdepth: quick coverage calculation for genomes and exomes. Bioinformatics.

[61] Jia KH, Wang ZX, Wang L, Li GY, Zhang W, Wang XL (2022). SubPhaser: a robust allopolyploid subgenome phasing method based on subgenome-specific k-mers. New Phytol.

[62] Marçais G, Kingsford C (2011). A fast, lock-free approach for efficient parallel counting of occurrences of k-mers. Bioinformatics.

[63] Quinlan AR, Hall IM (2010). BEDTools: a flexible suite of utilities for comparing genomic features. Bioinformatics.

[64] Bushnell B: BBMap. A fast, accurate, splice-aware aligner. In: Conference: 9th Annual Genomics of Energy & Environment Meeting, Walnut Creek, CA, March 17–20, 2014; United States. DE-AC02–05CH11231 2016–04–08: 2014: Medium: ED.

[65] Gel B, Díez-Villanueva A, Serra E, Buschbeck M, Peinado MA, Malinverni R (2015). regioneR: an R/Bioconductor package for the association analysis of genomic regions based on permutation tests. Bioinformatics.

[66] Nordberg H, Cantor M, Dusheyko S, Hua S, Poliakov A, Shabalov I (2013). The genome portal of the Department of Energy Joint Genome Institute: 2014 updates. Nucleic Acids Res.

[67] Emms DM, Kelly S (2019). OrthoFinder: phylogenetic orthology inference for comparative genomics. Genome Biol.

[68] Zhang R-G, Li G-Y, Wang X-L, Dainat J, Wang Z-X, Ou S, Ma Y, et al. TEsorter: an accurate and fast method to classify LTR-retrotransposons in plant genomes. Hortic Res. 2022, 9. 10.1093/hr/uhac017 PMC9002660 35184178

[69] Hufford MB, Seetharam AS, Woodhouse MR, Chougule KM, Ou S, Liu J (2021). De novo assembly, annotation, and comparative analysis of 26 diverse maize genomes. Science.

[70] Ou S, Su W, Liao Y, Chougule K, Agda JRA, Hellinga AJ (2019). Benchmarking transposable element annotation methods for creation of a streamlined, comprehensive pipeline. Genome Biol.

[71] Jukes TH, Cantor CR. CHAPTER 24 - evolution of protein molecules. In: Mammalian Protein Metabolism*.* Edited by Munro HN: Academic Press; 1969: 21-132.

[72] Chang X, He X, Li J, Liu Z, Pi R, Luo X, Wang R, Hu X, Lu S, Zhang X (2024). High-quality *Gossypium hirsutum* and *Gossypium barbadense* genome assemblies reveal the landscape and evolution of centromeres. Plant Commun.

[73] Wicker T, Gundlach H, Spannagl M, Uauy C, Borrill P, Ramirez-Gonzalez RH, Mayer KFX, Paux E, International Wheat Genome Sequencing C (2018). Impact of transposable elements on genome structure and evolution in bread wheat. Genome Biol.

[74] Kurtz S (2003). The Vmatch large scale sequence analysis software. Computer Program.

[75] RepeatMasker Open-4.0 [http://www.repeatmasker.org]

[76] Katoh K, Misawa K, Kuma Ki, Miyata T (2002). MAFFT: a novel method for rapid multiple sequence alignment based on fast Fourier transform. Nucleic Acids Res.

[77] He X, Qi Z, Liu Z, Chang X, Zhang X, Li J, et al. Pangenome analysis reveals transposon-driven genome evolution in cotton. figshare. 10.6084/m9.figshare.24354895 (2024). 10.1186/s12915-024-01893-2 PMC11040754 38654264

