# Supplementary material for: Pangenome analysis reveals transposon-driven genome evolution in cotton
Source: BMC Biol. 2024 Apr 23;22:92. doi: 10.1186/s12915-024-01893-2 (PMC11040754; doi:10.1186/s12915-024-01893-2)
Supplement: Supplementary file 1 — Additional file 1. Fig. S1. BUSCO statistics and NRSs alignment distribution. Fig. S2. Distribution of different classifications of genes in the reference genome and non-reference sequences. Fig. S3. The PCR experiment confirmed the five non-reference genes. Fig. S4. Gene feature comparisons between core, softcore, shell, and cloud genes. Fig. S5. GO enrichment for selected and unselected genes. Fig. S6. Schematic diagram illustrating the overall process of identifying the A2-nascent sequences and At-lost sequences. Fig. S7. Comparison of gene characteristics with different ages. Fig. S8. Gene age distribution in different single-copy gene status. Fig. S9. The LTR-RT amplification patterns between four genomes. Fig. S10. The lifespan of the LTR-RT cluster. Fig. S11. The adjacent homologous genes distance between diploid and tetraploid cotton. [file 12915_2024_1893_MOESM1_ESM.pdf]

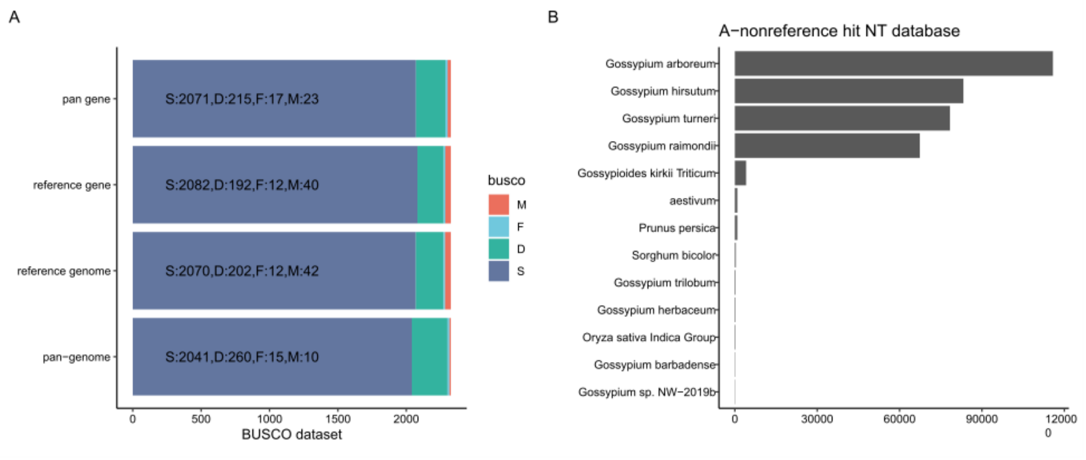


**Fig. S1.** (A) BUSCO analysis in the reference genome and pan-genome indicates that pan-genome assembly improved both genome sequences and genes, effectively reducing the number of missing BUSCO genes. (B)The non-reference sequence aligned to the NT database in NCBI.

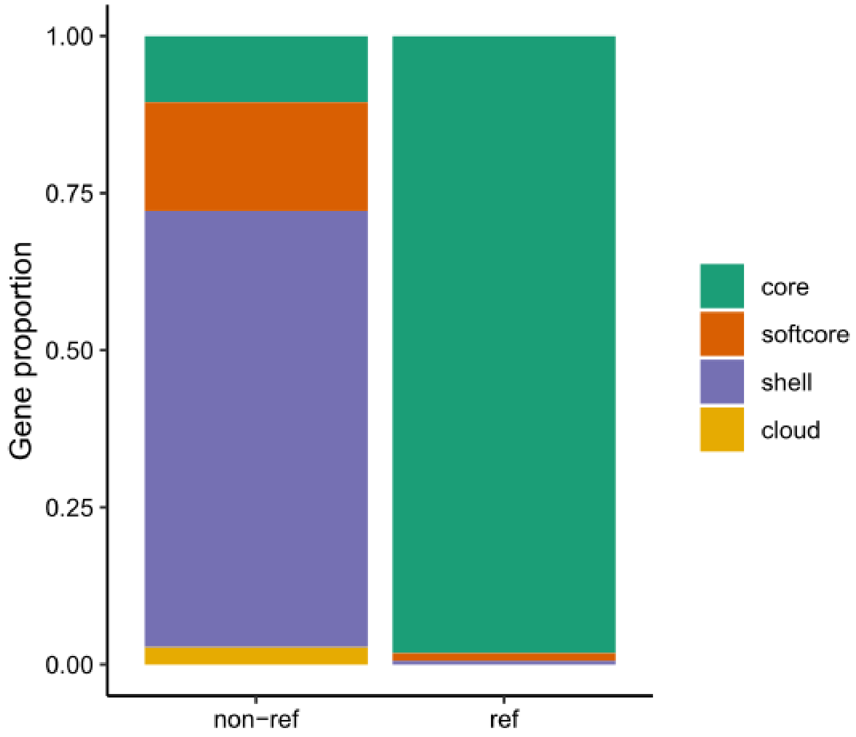


**Fig. S2.** Distribution of different classifications of genes in the reference genome and non-reference sequences.

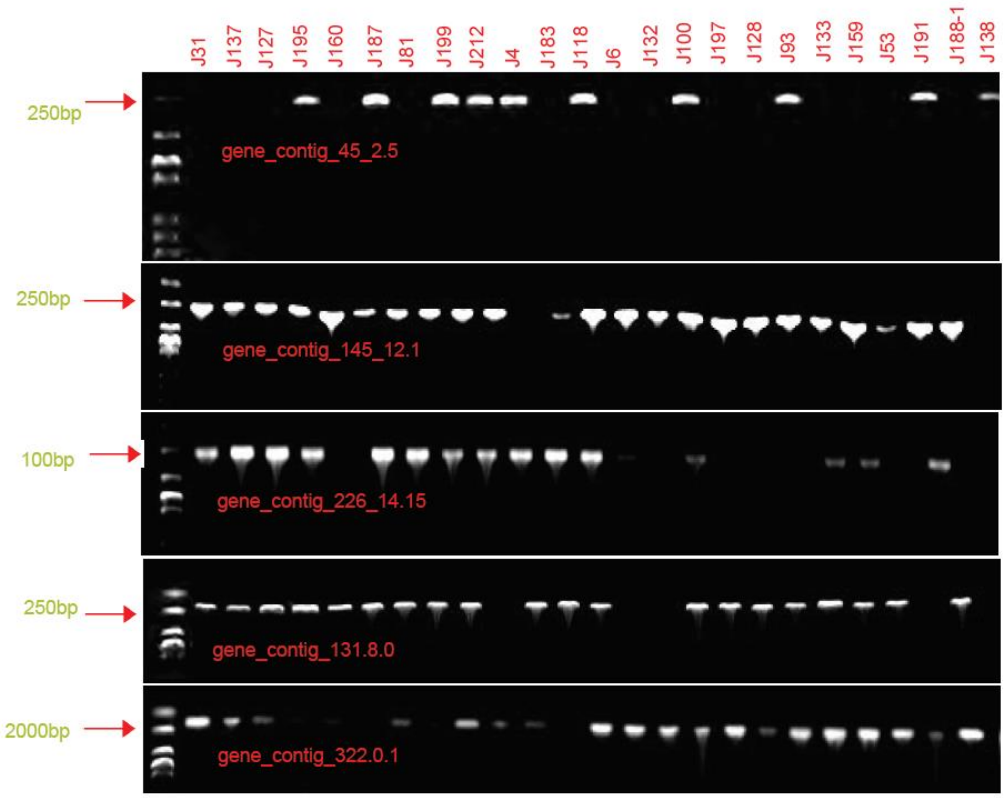


**Fig. S3.** The distribution of five random non-reference gene sequences in 24 accessions was verified by PCR. The size of the DNA markers is highlighted by red arrows.

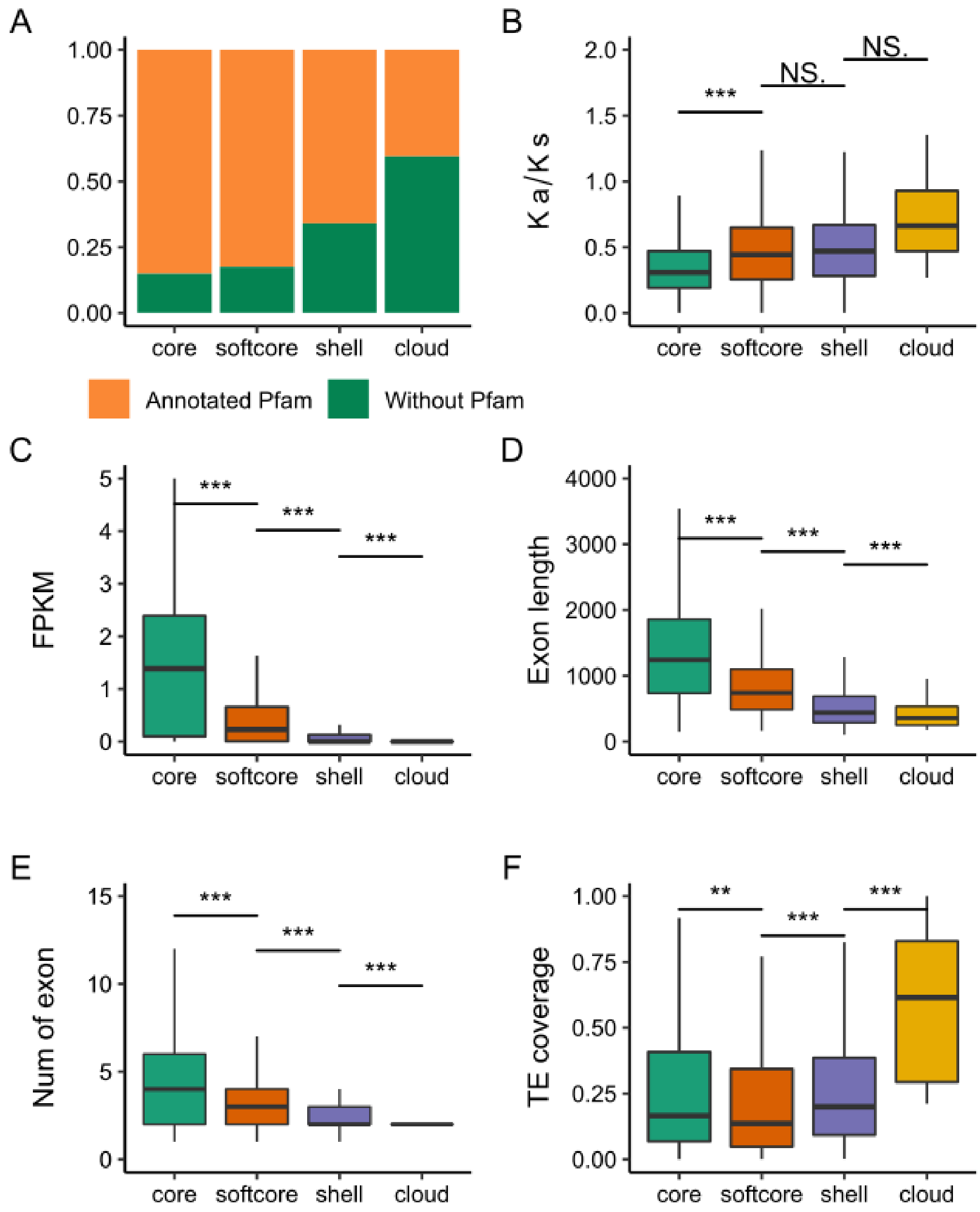


**Fig. S4.** Gene feature comparisons between core, softcore, shell, and cloud genes. (A) The proportion of genes with annotated Pfam domains in core, softcore, shell, and cloud genes. (B-F) The $K_a/K_s$ ratio, gene expression level, exon length, number of exons, and TE coverage in different classifications of genes.

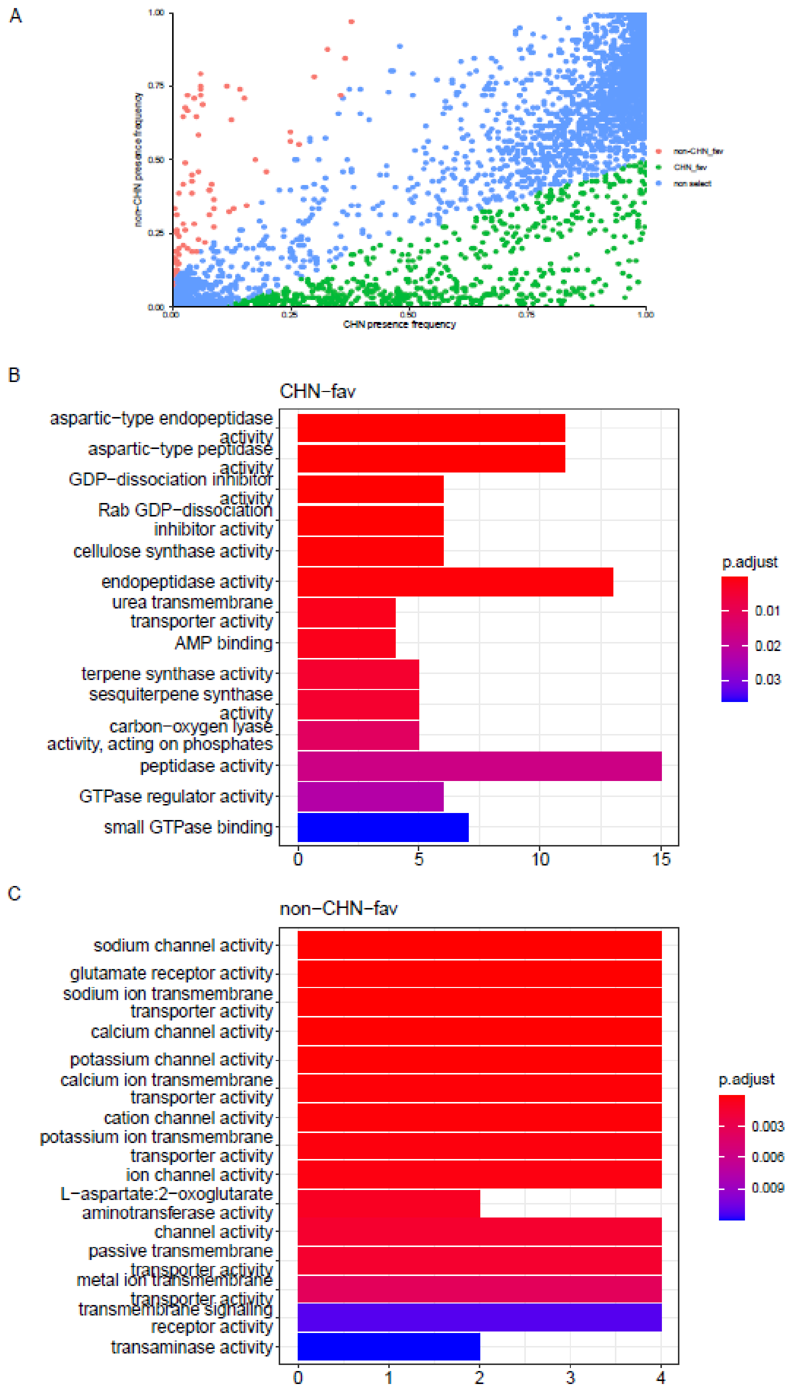


**Fig. S5.** GO enrichment for selected and unselected genes. (A) Comparison of significant gene presence frequency between China and abroad. (B) GO enrichment analysis of genes with copy number gain and loss during domestication.

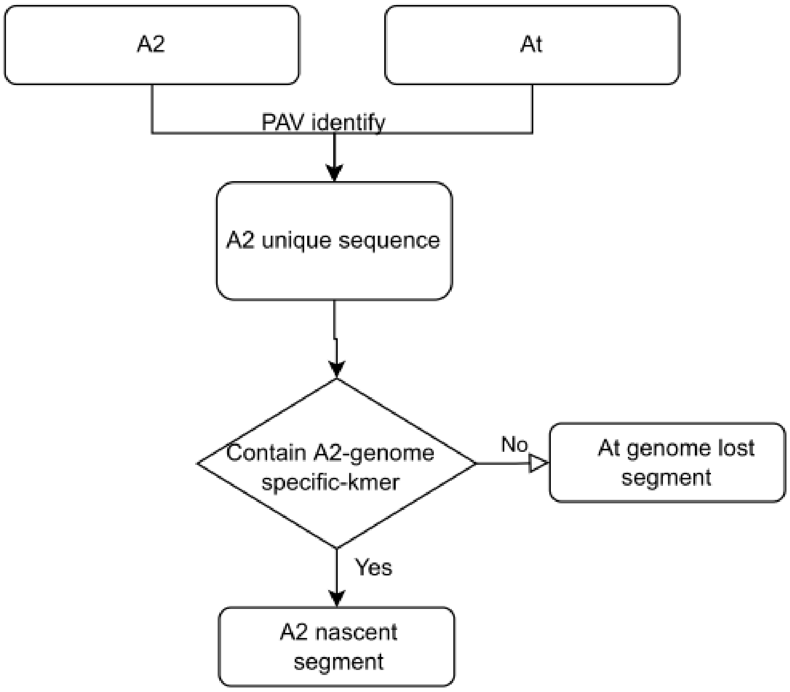


**Fig. S6.** Schematic diagram illustrating the overall process of identifying the $A_2$-nascent sequences and At-lost sequences.

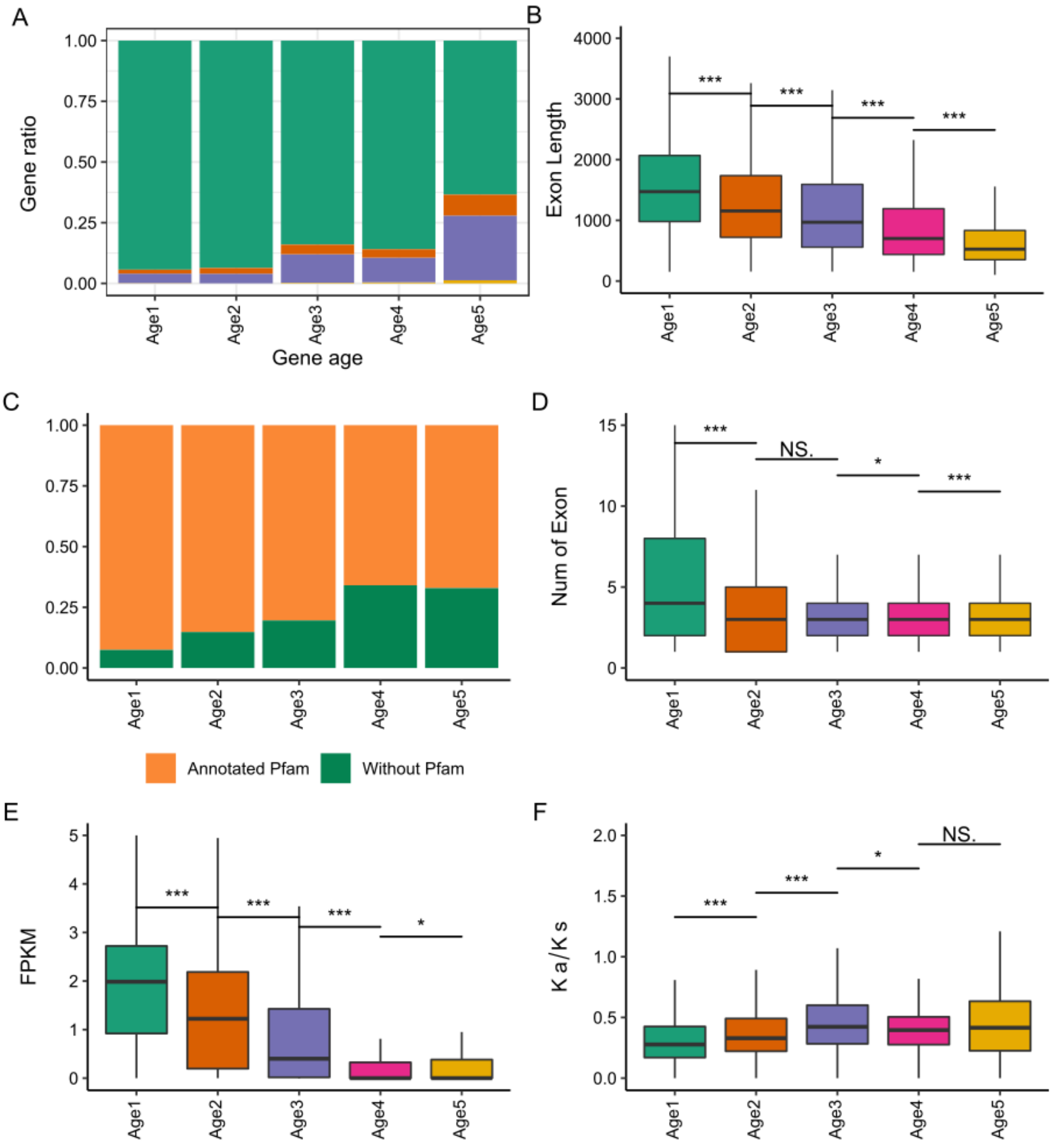


**Fig. S7.** Comparison of gene characteristics with different ages. (A) Gene conservation in populations is significantly correlated with gene age. (C-H) Similar to **Fig. S4**, comparison of genomic features of genes with different ages.

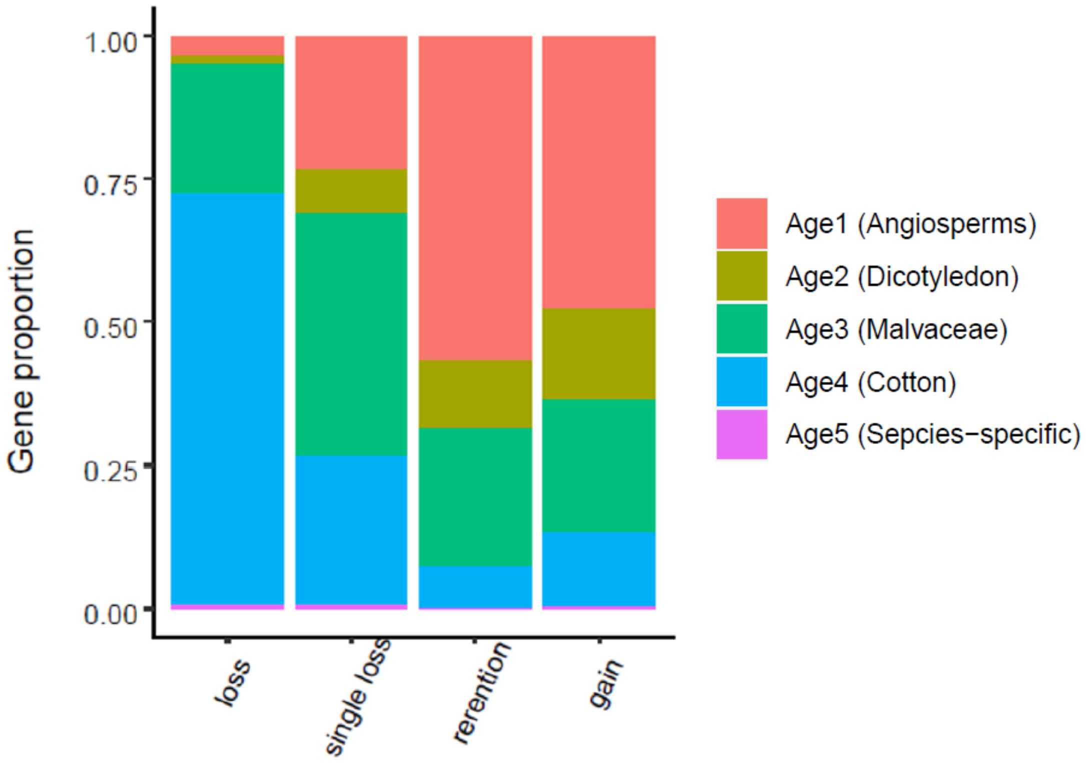


**Fig. S8.** Gene age distribution in different single-copy gene status. In polyploid cotton, gained genes exhibit greater conservation compared to lost genes.

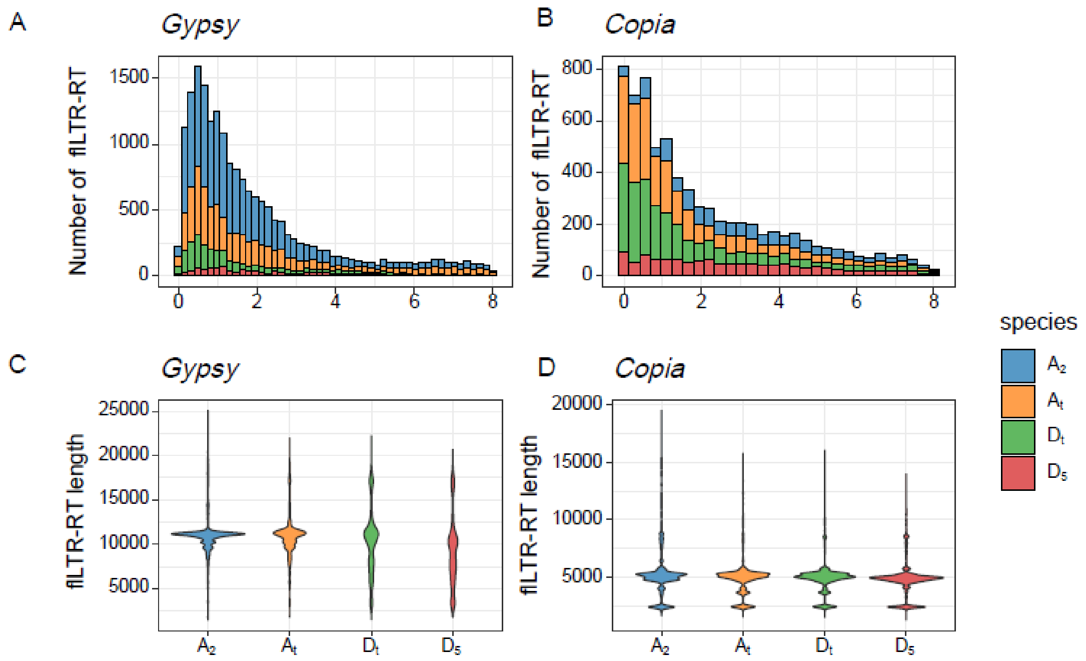


**Fig. S9.** The LTR-RT amplification patterns between four genomes. (A-B) Temporal pattern of flLTR-RT insertion in four cotton genomes. For *Gypsy*, only the D5 genome did not expand significantly after polyploidy, and the amount of $A_2$ is greater than At. For *Copia*, only two subgenomes of tetraploid showed amplification. The numbers on x-axis represent the LTR insertion time (Million years ago). (C-D) The length distribution of fl-LTR-RT. *Copia* is conserved among species, only $A_2$ has a large number of *Gypsy* with the length ~12000 bp.

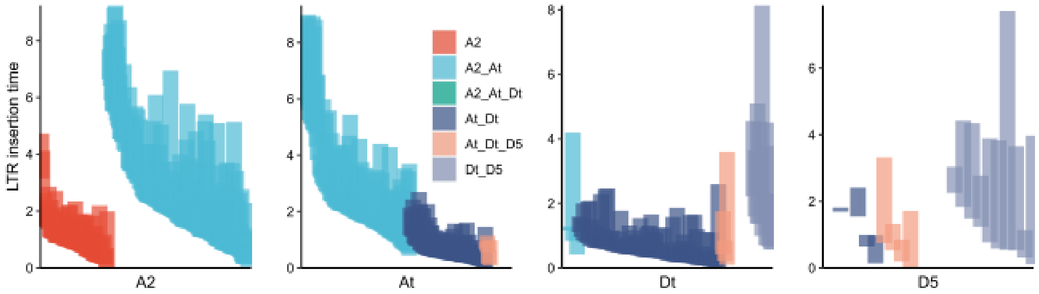


**Fig. S10.** The lifespan of the LTR-RT cluster. Lineages unique to the A or D subgenome occurred down to 2 million years ago, confirming the estimated time for tetraploidization. Moreover, both the At and Dt subgenomes showed a remarkable LTR concerted proliferation after polyploidization.

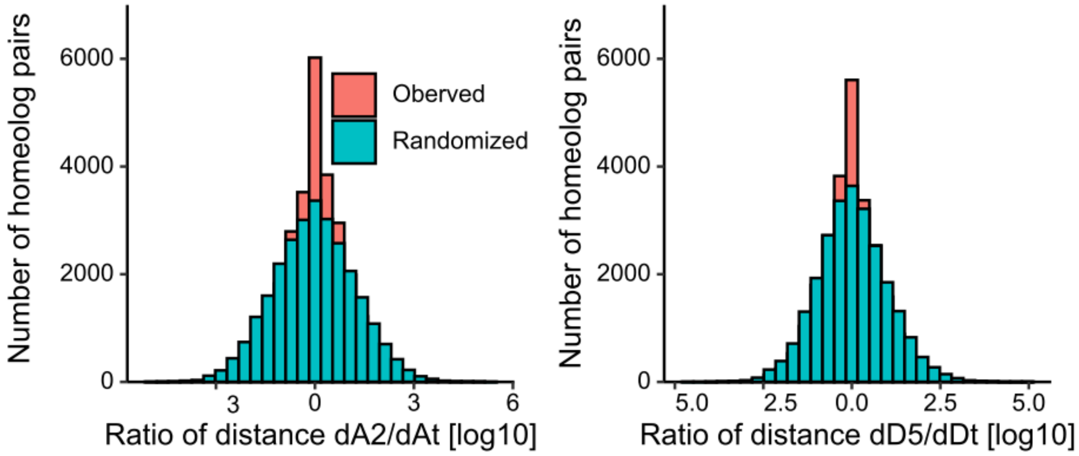


**Fig. S11.** The homologous genes from 356 gene synteny blocks were shared in four genomes. We compare the distance from each adjacent homologous gene in diploid and tetraploid, the distribution is compared with randomized gene position (see methods).
